# Looking back and looking forward

**DOI:** 10.1093/jxb/erab424

**Published:** 2021-09-25

**Authors:** John E Lunn

**Affiliations:** Journal of Experimental Botany

**It is just over a year since I succeeded Christine Raines as Editor-in-Chief of the *Journal of Experimental Botany*, a year that brought unprecedented challenges for all of us due to the Covid19 pandemic. Nevertheless, despite these challenges, I am pleased to report that the *Journal of Experimental Botany* has weathered the storm and seen a substantial rise in our Clarivate^TM^ Web of Science^TM^ impact factor to 6.992, reflecting the high quality of the papers published by the journal.**

This achievement is based on the strong global standing of the journal that was built up under Christine’s custodianship, and the dedication of the *Journal of Experimental Botany* editorial office staff and members of the editorial board, who have kept the journal running smoothly through these uncertain and difficult times. In the past year, we have expanded the editorial board to broaden our coverage in all areas of plant science that we publish and diversify the board to better reflect the global plant science community we serve. I would also like to take this opportunity to thank all of the authors who entrusted their work to us for publication, and express our sincere gratitude to all the reviewers who generously gave their time and expertise to support the journal by helping our authors to achieve the full potential of their work in published form.

The *Journal of Experimental Botany* was founded by the Society for Experimental Biology (SEB) in 1950 to publish high quality plant science, and remains a community-based journal under the aegis of the SEB to this day. We had planned to mark the journal’s 70^th^ anniversary with a special symposium at the 2020 SEB annual meeting, but unfortunately had to postpone and scale back our plans when that meeting was cancelled due to the pandemic. However, with fantastic support from the SEB events team, we ventured into the brave new world of virtual meetings by holding an online mini-symposium in December 2020, on the theme of: ‘*Photosynthesis in a Changing World*’. The presenters and other invited authors also contributed to a special 70^th^ anniversary issue of *Journal of Experimental Botany* on Plant Metabolism in a Changing World, which has just been published. Encouraged by the positive reception of the online symposium, we have initiated a series of JXB webinars together with the SEB, in which authors of selected *Journal of Experimental Botany* papers discuss the background and significance of their recently published work. Recordings of these webinars are freely available from the SEB website.

The last year has seen a number of changes in the format of the *Journal of Experimental Botany*. Reflecting a growing trend in submissions, we have added a new category of research paper – Technical Innovations and Community Resources – for authors to present new experimental methods or publicly available tools, databases or germplasm that will be of broad interest to the plant science community. In a related move, we no longer include the Materials and methods section in the manuscript word count, allowing authors to report a full description of their experimental work. In addition to research articles, *Journal of Experimental Botany* also publishes reviews, including our series of commissioned Darwin Reviews by eminent scientists, and we welcome suggestions from the community for potential review topics and authors. At the beginning of 2021, *Journal of Experimental Botany* moved to online-only publication, allowing greater efficiency in the publication of papers and better integration of data in non-traditional formats (e.g. videos). We thank the staff at our publisher, Oxford University Press, for helping to make this transition as seamless as possible. Papers are published online as soon as they are accepted, initially in manuscript form and then as corrected proofs, before final publication in one of our regular or special issues. We publish about ten Special Issues a year, often associated with a scientific meeting, and invite suggestions from the community for new topics.

Looking to the future, we have great pleasure in announcing that the *Journal of Experimental Botany* has become affiliated with Plant Direct, an open access, sound science journal (impact factor 3.038). *Plant Direct* was jointly founded by the SEB and American Society of Plant Biologists to publish work that had been considered for publication by the societies’ senior journals and judged to have merit, but which did not meet all the criteria for publication in those journals. Following our link with *Plant Direct*, authors now have the option to transfer their manuscript, along with the peer reviews and editorial decision, directly from the *Journal of Experimental Botany* to *Plant Direct*, allowing a rapid decision by the *Plant Direct* editors. Authors can indicate at the time of submission if they wish their manuscript to also be considered for publication by *Plant Direct*. Alternatively, our editors may suggest this option if a manuscript is not accepted for publication in the *Journal of Experimental Botany*. We believe the *Plant Direct* option offers our authors a more efficient route to publication and will also be appreciated by our reviewers, as this process will maximize the value of their voluntary service to the community.

